# Data from the Human Penguin Project, a cross-national dataset testing social thermoregulation principles

**DOI:** 10.1038/s41597-019-0029-2

**Published:** 2019-04-17

**Authors:** Chuan-Peng Hu, Ji-Xing Yin, Siegwart Lindenberg, İlker Dalğar, Sophia C. Weissgerber, Rodrigo C. Vergara, Athena H. Cairo, Marija V. Čolić, Pinar Dursun, Natalia Frankowska, Rhonda Hadi, Calvin J. Hall, Youngki Hong, Jennifer Joy-Gaba, Dušanka Lazarević, Ljiljana B. Lazarević, Michal Parzuchowski, Kyle G. Ratner, David Rothman, Samantha Sim, Cláudia Simão, Mengdi Song, Darko Stojilović, Johanna K. Blomster, Rodrigo Brito, Marie Hennecke, Francisco Jaume-Guazzini, Thomas W. Schubert, Astrid Schütz, Beate Seibt, Janis H. Zickfeld, Hans IJzerman

**Affiliations:** 1 Neuroimaging Center, Focus Program Translational Neuroscience (FTN), University Medical Center Mainz, Mainz, Germany; 2 Deutsches Resilienz Zentrum (DRZ), Mainz, Germany; 3 School of Psychology, Northwest Normal University, Lanzhou, China; 4 Department of Sociology & Interuniversity Center for Social Science (ICS), University of Groningen, Groningen, The Netherlands; 5 Department of Social Psychology, Tilburg University, Tilburg, The Netherlands; 6 Department of Psychology, Middle East Technical University, Cankaya, Ankara, Turkey; 7 Department of Psychology, Universität Kassel, Kassel, Germany; 8 Biomedical Neuroscience Institute, Faculty of Medicine, Universidad de Chile, Santiago, Chile; 9 Department of Psychology, Virginia Commonwealth University, Richmond, VA USA; 10 Faculty of sport and physical education, University of Belgrade, Belgrade, Serbia; 11 Department of Psychology, Afyon Kocatepe University, Afyonkarahisar, Turkey; 12 Center of Research on Cognition and Behavior, SWPS University of Social Sciences and Humanities, Sopot, Poland; 13 SWPS University of Social Sciences and Humanities, Warsaw, Poland; 14 Saïd Business School, University of Oxford, Oxford, UK; 15 Department of Psychological & Brain Sciences, University of California Santa Barbara, Santa Barbara, USA; 16 Institute of Psychology and Laboratory for research of individual differences, Faculty of philosophy, University of Belgrade, Belgrade, Serbia; 17 Nova School of Business and Economics, Universidade Nova de Lisboa, Carcavelos, Portugal; 18 Católica Research Centre for Psychological, Family and Social Well-Being & Católica-Lisbon School of Business and Economics, Universidade Católica Portuguesa, Lisbon, Portugal; 19 Department of Experimental and Applied Psychology, Vrije Universiteit Amsterdam, Amsterdam, The Netherlands; 20 Department of Psychology, Faculty of Philosophy, University of Belgrade, Belgrade, Serbia; 21 Department of Psychology, University of Oslo, Oslo, Norway; 22 HEI-Lab/School of Psychology and Life Sciences, Lusófona University, Lisbon, Portugal; 23 Department of Psychology, University of Siegen, Siegen, Germany; 24 Escuela de Psicología, Facultad de Ciencias Sociales, Pontificia Universidad Católica de Chile, Santiago, Chile; 25 Clínica Alemana de Santiago, Facultad de Medicina Clínica Alemana, Universidad del Desarrollo, Santiago, Chile; 26 Instituto Universitário de Lisboa (ISCTE-IUL), CIS-IUL, Lisboa, Portugal; 27 Faculty of Human Sciences and Education, University of Bamberg, Bamberg, Germany; 28 LIP/PC2S, Université Grenoble Alpes, Grenoble Alpes, France

**Keywords:** Human behaviour, Interdisciplinary studies

## Abstract

In *the Human Penguin Project* (*N* = 1755), 15 research groups from 12 countries collected body temperature, demographic variables, social network indices, seven widely-used psychological scales and two newly developed questionnaires (*the Social Thermoregulation and Risk Avoidance Questionnaire* (STRAQ-1) and the *Kama Muta Frequency* Scale (KAMF)). They were collected to investigate the relationship between environmental factors (e.g., geographical, climate etc.) and human behaviors, which is a long-standing inquiry in the scientific community. More specifically, the present project was designed to test principles surrounding the idea of *social thermoregulation*, which posits that social networks help people to regulate their core body temperature. The results showed that all scales in the current project have sufficient to good psychometrical properties. Unlike previous crowdsourced projects, this dataset includes not only the cleaned raw data but also all the validation of questionnaires in 9 different languages, thus providing a valuable resource for psychological scientists who are interested in cross-national, environment-human interaction studies.

## Abstract (summary)

**Table:** 

Design Type(s)	data collection and processing objective • behavioral data analysis objective
Measurement Type(s)	thermoregulation behavior
Technology Type(s)	crowd-sourced data generation
Factor Type(s)	age • sex • geographic location • Social Role
Sample Characteristic(s)	Homo sapiens • Germany • Chile • Turkey • Kingdom of Norway • United Kingdom • Poland • Portuguese Republic • Serbia • Singapore • China • United States of America • Switzerland

Machine-accessible metadata file describing the reported data (ISA-Tab format)

## Background & Summary

The relationship between environmental factors (e.g., geographical, climate etc.) and human behaviors is part of a long-standing scientific inquiry^1,2^, which has been long pursued by environmental psychologists^3^, among researchers from fields beyond psychology such as anthropology, sociobiology, and behavioral ecology^4–6^. In fact, even attachment theorist John Bowlby recognized that physical warmth is an important building block for attachment bonds^7^, while Harry Harlow launched the hypothesis of social thermoregulation in *The Nature of Love – Simplified*, describing a study where rhesus monkey infants with hot surrogate mothers were more explorative than those with physical cold surrogate mothers^8^. In recent times, psychologists have found, even with advances in modern ways to regulate temperature and other physical threats to survival, remnants of environmental influences on human behavior and cognition persist. For example, a recent study found that personality is correlated with (and likely influenced by) climate^9^. Similarly, the relationship between temperature and violence is an often-debated topic related to environment and human behavior^10,11^.

One particularly compelling phenomenon about environmental factors and animal behaviors (including humans) is a phenomenon called *social thermoregulation*. Like other homeothermic endotherms (warm-blooded animals that can regulate their temperature internally), humans also need to keep a relatively constant core body temperature to survive. Studies have shown that nonhuman homeothermic endotherms, like rodents^12^ and monkeys^13^, have developed social mechanisms to regulate their core body temperature. A positive correlation between the lower bound of core temperature in colder environments and social network size was found in vervet monkeys, a species often studied because of their similarity to humans^13^. Perhaps unsurprisingly, in humans, there is also a relationship between temperature and social bonds. For instance, temperature regulation is linked with trust (or psychological warmth)^14^. Beyond the bare biological mechanisms like huddling, Inagaki and colleagues found in a pilot study that feeling socially connected relates to higher core body temperature in humans^15^. Recent studies showed that the positive relationship between core body temperature and social network size may exist in humans^14,16^. Also, priming of (lack of) trust leads to higher temperature perceptions when temperatures are low, but not when temperatures are high^17,18^. Furthermore, pre-registered research also showed that feeling cold increases the need to socially connected^19^, and the accessibility of loved ones (especially for closer others)^20^.

As *social thermoregulation* related studies have expanded to diverse domains in the last decade^20^, some key issues have remained unexplored. For example, while Inagaki *et al*.’s pilot study shows a positive relationship between feelings of social connectedness and higher core body temperature^15^, it was previously unknown whether the social network buffers from the cold, and *which* variables predict this protection from the cold.

We thus identified – a priori – basic ideas to test social thermoregulation principles (i.e., does the social network protect against the cold?), but we did not have specific, priori predictions. To *generate* predictions from data, we relied on an inductive (as opposed to a deductive) approach, where we generated hypotheses from data using machine learning. We conducted a crowdsourced cross-national study, the Human Penguin Project (https://osf.io/2rm5b/, two studies, *N* = 1755), through a large-scale survey including self-reported psychological constructs and core body temperature measurements that participants recorded themselves^21^. The project consisted of an online pilot study (*N* = 232) and a large, cross-national study (15 research groups from 12 countries, *N* = 1523). In both studies, we measured a number of known correlates of core body temperature using a questionnaire and a number of social relationship variables that – based on prior research – should logically be related to core body temperature (e.g., nostalgia^22,23^ or attachment to homes^19^). We also included a number of variables that have been found to relate to body temperature in the medical literature (stress^24^; whether participants use medication or not e.g.^25^, and gender^26^) or to metabolism (like daily (diet) sugary drinks consumption^27^) and social network quality.

The principle that guided our selection of variables was over- rather than under-inclusive, therefore, we also asked questions that relate to the regulation of stress (and could thus relate to body temperature) like self-control^28^, attachment^29^, and access to one’s own feelings and bodily states (alexithymia^30^). See Table 1 for the total list of variables. Our inductive (i.e., data-driven) approach thus allowed us to generate hypotheses and predictions^31^. Using machine learning (more specifically, a Conditional Random Forest; CRF) in two datasets, we identified the most important predictors in our data. In our pilot study, we identified Complex Social Integration (CSI) as an important (positively related) predictor of Core Body Temperature (CBT).

**Table 1:** Variables in the Human Penguin Project.

Variable categories	Details
Body temperature	Oral temperature (measured twice)
Physiological information	Whether people used medication or not (and what kind)*
	Whether people smoke or not (and if so, how many cigarettes)
	Daily sugary drinks consumption (“gluctot”)
	Diet drinks consumption (“artgluctot”)^27^
Basic information	Birth year
	Gender
	Height*
	Weight*
	Sexual orientation*
	Whether people were in a romantic relationship or not,
	To what degree people identified themselves being monogamous,
	Self-reported health condition
	Language.
Location and whether	Minimum temperature of the day
	Average humidity of the day
	Distance from equator
	Longitude*
Social network	Three subscales of the Social Network Index, 32 items^34^: network size, embedded in the social network (socialembedded), complex social integration (CSI)
Established psychological scales	Trait Self Control Scale^28^ (13 items)
	Perceived Stress Scale^35^ (14 items)
	Southampton Nostalgia Scale (6 items about nostalgia and item about frequencies of nostalgia experience^22,23^)
	Home Attachment Scale (9 items from two sub-scales - home experience and rootness - of the place attachment scale by Harris *et al*. 1996^36^)
	Nomophobia questionnaire^37^ (attach to phone, items 1–9; online id, item 10–20)
	Experiences in Close Relationships Questionnaire-Revised (ECR-R)^29^, (anxiety, items 1–18; avoidance, items 19–36)
	Two subscales from the revised version of Toronto Alexithymia Scale (TAS-20)^30^: Difficulties in Identifying and Describing Feelings (DIDF), 11 items; Externally oriented thinking (EOT) 5 items
New scales	Social Thermoregulation and Risk Avoidance Questionnaire (STRAQ-1)^32^^#^
	Kama Muta Frequency Scale (KAMF)^38^

*These variables are not included in our open data to protect the identity of participants and are available upon request from the first author.

^#^See Vergara *et al*.^32^ for the final version of STRAQ-1.

Based on this, we conducted a second study, where we again found Complex Social Integration to be an important (and again positively related) predictor of Core Body Temperature. Because we had identified CSI as an important predictor of CBT in the pilot study, we also ran CRFs to identify predictors of CSI. This identified distance from the equator as a predictor of CSI.

We then reused the second dataset and, with the now generated hypotheses, we cross-validated a mediation model generated in our training set in a hold-out set. After all, given the large sample size of our dataset, we could randomly split the whole dataset, explore a mediation model in the first half of data to identify variables that could predict protection from the cold, then we cross-validated the mediation model in the other half of the data^21^.

In sum, this dataset includes demographic variables, body temperature, social network indices, people’s self-reported health, seven widely-used psychological scales, local temperature information (humidity and temperature, distance from the equator), and newly developed questionnaires^32^. Moreover, the questionnaires used in our study adapted into 9 languages via a standard translate-back-translate approach^33^. Our descriptive analyses of the pilot study and the individual sites demonstrated that these questionnaires have mostly sufficient to good psychometric properties (Cronbach’s alpha range from 0.71 to 0.98, except TAS-EOT with values as low as 0.21; McDonald’s omega range from 0.62~0.99, except TAS-EOT with a range of 0.48~0.76, see Online-only Table 1). Multi-site indices showed largely good psychometric properties (Cronbach’s alpha range from 0.83~0.93, except for TAS-EOT with 0.51; McDonald’s omega range from 0.62~0.96, except for TAS-EOT with 0.61; see Online-only Table 2).

## Methods

### Ethics and Consent

This research was approved under an “umbrella” ethics proposal at Vrije Universiteit, Amsterdam and at each site where there was a local ethics board (all ethics approvals can be found at the project page from the individual site: https://osf.io/2rm5b/). This study complied with ethics code outlined in the Declaration of Helsinki.

### Pilot Study

The main goal of the pilot study was to provide a proof-of-concept (i.e., providing evidence that the research protocol worked), so that the potential collaborators can saw the value of participating.

### Participants

The pilot study included participants from Amazon Mechanical Turk (mTurk, https://www.mturk.com/, *N* = 143) and Prolific Academic (https://prolific.ac/, *N* = 148) in 2015. Participants were requested to complete the survey between 9–11am, not to eat or drink anything warm or cold for 10 minutes preceding the survey, and not to have exercised an hour preceding the survey. Because the sample was relatively small, we excluded all those participants that did not adhere to these guidelines (mTurk, *N* = 3; Prolific Academic, *N* = 56), which left 232 remaining participants.

### Procedure

**Fig. 1:**
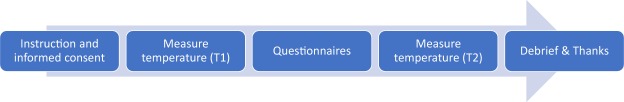
The general flow of the survey. Participants first read the instructions and informed consent, after agreed to proceed, they filled out the questionnaire battery, and measured their oral temperature before and after these questionnaires. For both oral temperature measurements, they submitted a photo of their thermometer (and wrote down measurement (1 or 2) and the date).

Participants entered the survey, where they were requested to fill in a number of different questionnaires (see Fig. 1). At the beginning of the questionnaire (Measurement 1) and at the end of the questionnaire (Measurement 2), they were requested to measure their own oral temperature with an oral thermometer, take a picture of the thermometer (with date, time, and Measurement– 1 or 2 – included as a note in the picture).

Between two measurements of core temperature, participants answered a series of questionnaires, including the social network index questionnaire (“networksize”, “socialembedded”, and a measure on complex social integration, “CSI”)^34^. CSI includes an inventory of the following ties: Relationships with spouse, parents, parents-in-law, children, other close family members, neighbors, friends, workmates, schoolmates, fellow volunteers (e.g., charity or community work), members of groups without religious affiliations (e.g., social, recreational, professional), and members of religious groups. One point was assigned for participation in each kind of relationship for which respondents reported that they spoke (in person or on the phone) to someone in that relationship at least once every 2 weeks.

Other scales include the Perceived Stress Scale (variable named as “stress”)^35^, the Southampton Nostalgia Scale (“nostalgia”)^22^, people’s attachment to homes (“attachhome”)^36^, people’s attachment to their smartphone (often referred to as nomophobia)^37^, daily sugary drinks consumption (“gluctot”) and diet drinks consumption (“artgluctot”)^27^, self-control (“selfcontrol”)^28^, attachment in close relationships (divided into “avoidance” and “anxiety”)^29^, and access to one’s own feelings and bodily states (alexithymia; divided into subscales externally oriented thinking (“EOT”) and difficulty identifying one’s feelings (“DIDF”))^30^. The dataset also includes information about participants’ sex (“sex”), height (“height”), weight (“weightkg”), and whether they used medication (“meds”). Finally, we looked up the minimum temperature (“mintemp”) and average humidity of the day (“avghumidity”) based on participants’ IP address by using a weather history site (http://www.wunderground.com/history/), which bases weather on the nearest airport.

At the end of the survey, participants were thanked for their participation and debriefed.

## Cross-National Data

### Participants

The confirmatory dataset was collected in various countries (*N* = 12); 1536 participants finished the survey in 2016. We reviewed all pictures that participants uploaded to our Qualtrics platform, and made 193 (mostly small) corrections to the CBT values, based on pictures participants uploaded. When no picture was uploaded, we kept the participant in our dataset. When generic pictures or pictures that were irrelevant for our study were uploaded, we deleted the participant (*N* = 12). We also deleted participants from our dataset that had identical value and IP address as other participants (*N* = 1). Our final sample consisted of 1523 participants.

### Procedure

We first created one central survey in English. For the non-English speaking site, we then translated and back-translated the survey keeping in mind loyalty to the original meaning. The original and back-translated versions were checked to ensure meaning was comparable across versions. All surveys were then programmed into the online survey platform Qualtrics. Surveys were run online or in the lab across our different sites. Participants were requested to complete the survey between 9–11am in their local time zone, not to eat or drink anything warm or cold for 10 minutes preceding the survey, and not to have exercised an hour preceding the survey (To be sure, we also asked whether they did eat or drink anything warm or cold 10 minutes before the study (“eatdrink”) or whether they did exercise an hour preceding the study (“exercise”)). Participants entered the survey, where they were requested to fill in a number of different questionnaires. Before the first questionnaire (Measurement 1), we requested participants to measure their own oral temperature with an oral thermometer, which was repeated at the end of the questionnaire (Measurement 2). For both measurements, we asked to take a photograph of their thermometer and upload it to Qualtrics (with date, time, and whether it was Measurement – 1 or 2 – included on a note).

We used the same questionnaires as our pilot study, but now added a few questions that may also bear relevance for complex social integration and core body temperature, like whether people are in a romantic relationship or not (“romantic”), how monogamous they perceive themselves to be (“monogamous”), attachment to their smartphone and their online identity (often referred to as nomophobia; “attachphone” and “onlineid”)^37^, and we recorded participants’ longitude and latitude via a standard option available in Qualtrics (“longitude”; we calculated latitude into distance from the equator “DEQ”). For privacy reason, in the open dataset, we didn’t report longitude information (“longitude”).

Two other exploratory questionnaires were also included: the Kama Muta Frequency Scale (KAMF), which measures the frequency of participant have the feeling of being moved (Kama Muta)^38^, and the *Social Thermoregulation and Risk Avoidance Questionnaire* (STRAQ-1)^32^, which included 57 items and were measure for exploring the relationship between physical need of thermoregulation and adult attachment.

At the end of the survey, participants were thanked and debriefed for their participation. We again looked up the minimum temperature of that day and average humidity of that day through their IP address and the weather history site.

## Data Records

All data records listed in this section are available from the Open Science Framework (OSF)^39^ and can be downloaded without an OSF account. The datasets were anonymized to remove any information that can identify the participant responses, such as the identification number from Amazon’s Mechanical Turk, weight, height, sexual orientation, medication, and longitude. The R script used for pre-processing the semi-raw data is also available.

### All Datasets

Location: Open Science Framework^39^.

File format: comma separated values file (.csv).

These files contain: cleaned raw data, codebooks for each raw data.

### Questionnaires

Location: Open Science Framework^39^.

File format: Microsoft Word (.docx).

## Technical Validation

To validate that the scales used in different countries have good psychometrical properties, we used the multi-site data to calculate the indices of reliability for each scale per site. More specifically, we reported Cronbach’s alpha, McDonald’s omega (hierarchical and total)^40^. The results showed that the reliabilities for most scales used here are satisfying (see, Online-only Table 2). Thus, these data are good for further re-use.

## Usage Notes

Our main research – discovering which variables related to social connections best predict the protection of core body temperature – revealed that Complex Social Integration (CSI), defined as the number of types of high-contact roles one engages in, is a critical predictor of core body temperature^21^. The pilot study identified CSI as one of the most important predictors of CBT, after weight, height, and sex of participants, and the minimum temperature of that day. A cross-validation approach testing a mediation model further revealed that being further from the equator (as a proxy of being in a colder climate) relates to higher levels of CSI, which in turn relates to higher CBT^21^. Despite that the data are cross-sectional, we think that our theoretical principles are strong enough to infer that CSI forms a buffer from living in a colder climate. Furthermore, we also found that the effect held for those in romantic relationships (and not for those without). We don’t have a good explanation for the latter effect, and this is an important question for future studies. Interestingly, we also found other variables can predict the protection from the cold. For example, culture (language family) could potentially play a role that may be even more important than CSI^21^, opening up the door to investigate interrelationships between socio-economic development, level of close-knittedness in cultures^41^, and linguistic structures^42^ with complex social integration and temperature regulation. Nevertheless, the culture variable may simply be a measurement artifact and is thus an important avenue for future research.

Because the data are cross-sectional, causality needs to be better determined in future research. In addition, although we found robust relationships, it is also crucial to determine the proximal mechanisms that explain the protection of CBT in humans. The most promising avenue for future research to detect the relationship is the examination of social variables and peripheral temperature. The peripheral temperature, for example, drops after a brief episode of social exclusion^43^ and it is generally accepted as a defense mechanism against changes in the core as a result of fluctuations in ambient temperature^44^. For that reason, we think that the experience sampling of social experiences and moment-to-moment changes in peripheral temperature is the most promising route to discover socially-based temperature defense mechanisms. Because of rapid improvements in sensor technology, we have been able to develop software for smartphones to record moment-to-moment peripheral temperature fluctiations^45^. These projects should rely on similar data-driven approaches as we utilized here.

Beyond the DEQ-CBT-CSI model generated from this study, data from our main study have already been used in other projects. Van Lange and colleagues 2017, for example, proposed that distance from the equator shapes people’s ability to control themselves^10,11^. They stated that “lower temperatures and especially greater seasonal variation in temperature call for individuals and societies to adopt … a greater degree of self-control”. In developing their theoretical position, the authors proposed distance from the equator as a predictor of self-control. They advocated a “data-driven” approach, allowing one “to derive precise estimates of the variance accounted for by various predictor variables”. As trait self-control score^28^ was included in this dataset, we were able to test this proposition. Using the same supervised machine learning as in our main study, we found the distance from the equator to be a predictor of self-control but barely so: it was the 14^th^ predictor in our list and comparable to whether participants spoke Serbian or not^11^. All-in-all, the current dataset, and a data-driven approach could thus be used to reject Van Lange *et al*.’s approach that distance from the equator is an important factor in shaping self-control.

A third article was also written on items inserted in the dataset, again relying on split-half validation. This article concerned the development of the *Social Thermoregulation and Risk Avoidance Questionnaire* (STRAQ-1)^32^. The STRAQ-1 is another important step in discovering social-thermoregulation related principles in humans. Using a robust bootstrapping method, Vergara and colleagues identified 23 (out of 57) items in 4 subscales, which were named Social Thermoregulation (Omega = 0.83), High Temperature Sensitivity (0.83), Solitary Thermoregulation (0.77), and Risk Avoidance (0.57; see also Online-only Table 2). Because the dataset was rich in additional measures, it also allowed Vergara and colleagues to discover robust relationships between the STRAQ-1 and constructs often found to relate very closely with attachment (e.g., self-control, alexithymia, stress, and health). These results suggested that social-thermoregulation and risk-avoidance related desires may be crucial for (mental) health.

Please note that the reliabilities of most questionnaires in our data were stable across the samples we tested in different countries. For those established psychological scales, all of them have a relatively low cross- sample variability on reliability (SD of omega <= 0.08), except the Externally oriented thinking (EOT) subscale of Toronto Alexithymia Scale (TAS-20)^30^ (mean of omega = 0.63, SD = 0.1, from 0.48~0.71). This result, however, was consistent with a previous view that the TAS-20 should be used in combination with other instruments for clinical purpose^30^. As for the newly developed scales, the Kama Muta Frequency Scale (KAMF)^38^ has low cross-sample variability; while the STRAQ-1 has relatively high cross-nation variability for the Risk Avoidance subscale^32^. Therefore, when re-using the dataset, researchers should be mindful of this the cross-sample variability.

In short, the data we reported already allowed the validation of various well-known scales, which will allow considerable reuse for the generation of novel hypotheses, while the translated questionnaires will also be of considerable use for other cross-country comparison studies. Together, the dataset and materials shared will provide an important source for researchers who are interested in cross-national, human-environment interaction studies more generally, and those interested in social network, perceived stress, trait self-control, nostalgia, home attachment, nomophobia, experiences in close relationships, alexithymia, social thermoregulation, and being moved (Kama Muta) more specifically.

To promote the re-use of our dataset, we shared the summary data, the cleaned raw data, as well as the scripts used to go from raw to summary data, so that it is maximally useful for colleagues with different research goals. The raw data are accompanied by codebooks, which contain the information necessary for understanding the mapping between the column names in the raw data and the items of questionnaires, as well as how these items were scored. Finally, the questionnaires are available in 9 languages, providing a rich source for researchers who hope to collect data across nations (data that may identify participants (height, weight, et cetera) are available upon request from the first author).

### ISA-Tab metadata file

Download metadata file

## Data Availability

All the R scripts for calculating the reliability of the scales used in the current datasets were available on OSF^39^. We licensed the data under an Attribution-NonCommercial 4.0 International Creative Commons (CC BY-NC 4.0) license. The R packages used for processing the data were version 3.4.0 (2017-04-21). The packages used in generating current results as following: “tidyverse” (https://www.tidyverse.org/), “psych” (https://personality-project.org/r/psych/), and “foreign” (https://cran.r-project.org/package=foreign).

## Online-only Tables

**Online-only Table 1:** Participant demographics and reliabilities of questionnaires in the pilot study. N = number of participants

					Trait Self Control		Perceived Stress Scale		Nomophobia (with phone)		Nomophobia (onlineid)		ECR-R		ECR-R-Anxiety		ECR-R-Avoidance		Southampton Nostalgia Scale		TAS-DIDF		TAS-EOT		Home Attachment Scale	
**Site**	**Language**	**N**	**Female (%)**	**Birth year**	**α**	**ω**_**t**_	**α**	**ω**_**t**_	**α**	**ω**_**t**_	**α**	**ω**_**t**_	**α**	**ω**_**t**_	**α**	**ω**_**t**_	**α**	**ω**_**t**_	**α**	**ω**_**t**_	**α**	**ω**_**t**_	**α**	**ω**_**t**_	**α**	**ω**_**t**_
mTurk	English	140	64.3%	1964.2 (7.2)	0.88	0.91	0.82	0.93	0.90	0.93	0.91	0.94	0.97	0.98	0.95	0.97	0.96	0.97	0.95	0.98	0.91	0.94	0.58	0.75	0.88	0.93
Prolific Academic	English	92	59.8%	1967.5 (6.3)	0.88	0.91	0.85	0.93	0.87	0.91	0.90	0.94	0.95	0.97	0.94	0.95	0.95	0.96	0.95	0.98	0.91	0.94	0.55	0.69	0.91	0.95

**Online-only Table 2:** Site information, participant demographics, and reliabilities of questionnaires in the cross-national study. N = number of participants

						Trait Self Control		Perceived Stress Scale		Nomophobia (with phone)		Nomophobia (onlineid)		ECR-R		ECR-R-Anxiety		ECR-R-Avoidance		Southampton Nostalgia Scale		TAS-DIDF		TAS-EOT		Home Attachment Scale		STRAQ-1 (High Temperature Sensitivity)		STRAQ-1 (Social Thermoregulation)		STRAQ-1 (Solitary Thermoregulation)		STRAQ-1 (Risk Avoidance)		Kama Muta Frequency Scale	
**Country**	**Site**	**Language**	**N**	**Female (%)**	**Birth year**	**α**	**ω**_**t**_	**α**	**ω**_**t**_	**α**	**ω**_**t**_	**α**	**ω**_**t**_	**α**	**ω**_**t**_	**α**	**ω**_**t**_	**α**	**ω**_**t**_	**α**	**ω**_**t**_	**α**	**ω**_**t**_	**α**	**ω**_**t**_	**α**	**ω**_**t**_	**α**	**ω**_**t**_	**α**	**ω**_**t**_	**α**	**ω**_**t**_	**α**	**ω**_**t**_	**α**	**ω**_**t**_
Germany	University of Bamberg	German	42	69.0%	1982.1 (14.7)	0.81	0.87	0.92	0.94	0.89	0.87	0.92	0.95	0.96	0.97	0.95	0.96	0.93	0.95	0.90	0.96	0.91	0.89	0.48	0.69	0.86	0.91	0.85	0.90	0.73	0.85	0.70	0.81	0.62	0.64	0.85	0.93
Chile	University of Chile	Spanish	35	62.9%	1979.3 (13.2)	0.79	0.85	0.80	0.84	0.81	0.87	0.87	0.91	0.92	0.96	0.86	0.89	0.94	0.96	0.95	0.97	0.87	0.89	0.43	0.69	0.74	0.85	0.87	0.92	0.76	0.88	0.31	0.62	0.82	0.82	0.85	0.93
Germany	University of Kassel	German	106	69.8%	1990.3(7.8)	0.84	0.87	0.85	0.90	0.87	0.91	0.90	0.94	0.94	0.96	0.93	0.94	0.91	0.95	0.94	0.97	0.86	0.90	0.43	0.57	0.90	0.93	0.74	0.81	0.73	0.81	0.68	0.77	0.46	0.55	0.84	0.91
Turkey	Middle East Technical University	Turkish	181	65.7%	1992.4 (5)	0.83	0.86	0.89	0.91	0.89	0.91	0.86	0.93	0.93	0.95	0.90	0.92	0.92	0.94	0.94	0.97	0.89	0.92	0.51	0.60	0.92	0.95	0.78	0.89	0.73	0.80	0.77	0.85	0.52	0.54	0.86	0.93
Norway	University of Oslo	Norwegian	85	69.4%	1992.3 (6.4)	0.81	0.85	0.87	0.91	0.84	0.87	0.87	0.91	0.95	0.96	0.90	0.92	0.94	0.95	0.94	0.96	0.86	0.90	0.70	0.76	0.87	0.91	0.79	0.85	0.66	0.79	0.70	0.79	0.60	0.69	0.87	0.91
UK	University of Oxford	English	137	56.2%	1985.4 (13.5)	0.83	0.86	0.83	0.87	0.92	0.94	0.93	0.90	0.94	0.96	0.93	0.95	0.94	0.96	0.93	0.97	0.86	0.89	0.57	0.73	0.89	0.94	0.81	0.88	0.78	0.84	0.73	0.82	0.50	0.51	0.88	0.93
Poland	SWPS University of Social Sciences and Humanities	Polish	136	86.8%	1986.2 (8.9)	0.86	0.88	0.87	0.90	0.90	0.92	0.91	0.94	0.90	0.97	0.88	0.97	0.91	0.93	0.95	0.98	0.90	0.91	0.38	0.48	0.92	0.95	0.82	0.87	0.69	0.76	0.64	0.74	0.56	0.60	0.88	0.93
Portugal	University of Lusófona	Portuguese	21	33.3%	1984.1 (11.9)	0.71	0.85	0.76	0.85	0.94	0.91	0.88	0.93	0.87	0.97	0.86	0.97	0.74	0.95	0.98	0.98	0.80	0.91	0.21	0.48	0.89	0.94	0.71	0.84	0.54	0.85	0.59	0.74	0.55	0.73	0.90	0.94
Serbia	University of Belgrade	Serbian	164	80.5%	1993.7 (4.9)	0.85	0.88	0.88	0.91	0.91	0.94	0.90	0.93	0.93	0.97	0.90	0.92	0.93	0.96	0.94	0.98	0.89	0.92	0.57	0.69	0.90	0.93	0.76	0.85	0.80	0.84	0.73	0.80	0.58	0.59	0.88	0.93
Singapore	Singapore Management University	English	137	56.2%	1993.8 (1.5)	0.83	0.86	0.82	0.87	0.86	0.90	0.91	0.94	0.95	0.96	0.94	0.96	0.94	0.95	0.93	0.96	0.89	0.92	0.51	0.75	0.96	0.97	0.76	0.85	0.82	0.88	0.53	0.70	0.55	0.56	0.88	0.93
UK	University of Southampton	English	6	50.0%	1992.2 (1.6)	0.83	0.99	/	/	0.48	0.90	0.77	0.94	0.97	0.96	0.97	0.96	0.98	0.95	0.98	0.96	0.80	0.92	0.59	0.75	0.70	0.97	0.93	0.99	0.92	0.95	0.89	0.98	0.56	0.90	0.74	0.93
China	Tsinghua University	Mandarin	174	62.6%	1993.7 (6.4)	0.72	0.76	0.80	0.87	0.82	0.88	0.87	0.90	0.88	0.91	0.88	0.90	0.87	0.90	0.76	0.85	0.82	0.85	0.38	0.50	0.91	0.62	0.50	0.68	0.72	0.81	0.42	0.63	0.28	0.44	0.82	0.94
US	University of California, Santa Barbara	English	108	68.5%	1995.8 (1.7)	0.85	0.88	0.82	0.88	0.87	0.91	0.90	0.94	0.93	0.95	0.94	0.96	0.92	0.94	0.94	0.97	0.90	0.92	0.54	0.68	0.90	0.94	0.80	0.88	0.86	0.90	0.66	0.78	0.50	0.54	0.86	0.93
US	Virginia Commonwealth University	English	151	78.8%	1992.6 (4.7)	0.84	0.87	0.84	0.88	0.86	0.90	0.90	0.93	0.96	0.97	0.94	0.96	0.95	0.96	0.95	0.97	0.90	0.91	0.48	0.48	0.90	0.94	0.83	0.88	0.78	0.82	0.63	0.75	0.63	0.64	0.89	0.94
Switzerland	University of Zürich	German	40	72.5%	1987.6 (8.7)	0.79	0.87	0.82	0.90	0.86	0.91	0.87	0.93	0.95	0.97	0.95	0.97	0.91	0.95	0.96	0.98	0.88	0.91	0.60	0.57	0.90	0.94	0.89	0.93	0.72	0.80	0.75	0.86	0.70	0.78	0.85	0.94
	**Total (multi-site)**		1523	68.8%	1990.9 (8.4)	0.83	0.85	0.84	0.88	0.89	0.91	0.90	0.93	0.93	0.96	0.92	0.93	0.92	0.94	0.92	0.96	0.88	0.92	0.51	0.62	0.90	0.94	0.77	0.83	0.77	0.83	0.68	0.77	0.57	0.57	0.87	0.91
